# Metabolic Syndrome and Incidence of Laryngeal Cancer: A Nationwide Cohort Study

**DOI:** 10.1038/s41598-018-37061-0

**Published:** 2019-01-24

**Authors:** Sang-Yeon Kim, Kyung-do Han, Young-Hoon Joo

**Affiliations:** 1 Department of Otolaryngology-Head and Neck Surgery, College of Medicine, The Catholic University of Korea, Seoul, Korea; 2 Department of Biostatistics, College of Medicine, The Catholic University of Korea, Seoul, Korea

## Abstract

It is unknown whether the presence of metabolic syndrome (MetS) affects the incidence of laryngeal cancer. The aim of this national population-based retrospective study was to analyze the relationship between MetS and the incidence of laryngeal cancer. Patients with laryngeal cancer (ICD-10: C32) between 2009 and 2010 were retrospectively identified and tracked until 2015 using the Korean Health Insurance claims database. During the seven-year follow-up period, 5,322 subjects were newly diagnosed with larynx cancer. The mean age of people with laryngeal cancer was much higher than those without (63.29 vs. 47.7 years, p < 0.0001), and the incidence of larynx cancer in men was much higher than that in women (93.16% vs. 6.84%, p < 0.0001). Age, gender, smoking status, alcohol intake, and exercise-adjusted hazard ratios indicated that participants with MetS had a 1.13-fold higher hazard of having larynx cancer than those without MetS. The number of MetS components was a strong risk factor for laryngeal cancer with a higher risk estimate of this cancer in both ex- and current smokers as well as people who have never smoked. MetS was found to be an independent risk factor for the incidence of laryngeal cancer. In Korea, MetS and its components are significantly associated with the development of laryngeal cancer.

## Introduction

Anually, nearly 10,000 people are newly diagnosed as laryngeal cancer which is one of the most common cancer of the upper aerodigestive tract^1^. Smoking and alcohol use are supposed to be plausible etiological risk factors for laryngeal cancer. In recent years, metabolic syndrome (MetS) is associated with an increased risk of the development or progression of cancer. The prevalence of MetS has increased worldwide, in parallel with the increasing cancer incidence^2,3^.

MetS is strongly associated with the cancer risk and mortality. Abdominal obesity is thought to be a major risk factor for cancer; it is significantly linked to higher risk of most common cancers and higher mortality rates^4^. Diabetes mellitus is also closely related with risks of cancer incidence^5,6^. MetS has been shown to be associated with increased incidences of prostate cancer, colorectal cancer, and the recurrence of breast cancer^7–10^. In patients with MetS, hormonal disturbances are usually detected, and this hormonal deregulation is presumed as a risk factor for carcinogenesis in prostate, colorectal, and endometrial cancers^11–13^. However, there has not yet been any investigation conducted to reveal the association between MetS and laryngeal cancer. Thus, we evaluated the association of larynx cancer with MetS and its individual components in the Korean population.

## Results

### Basic characteristics

The characteristics of the study population are summarised in Table 1. During the seven-year follow-up period, 5,322 subjects were newly diagnosed with larynx cancer. The mean age of people with laryngeal cancer was much higher than those without (p < 0.0001), and the incidence of larynx cancer in men was much higher than that in women (p < 0.0001). The percent of participants who were either current or ex-smokers and heavy drinkers was significantly higher among those with larynx cancer than among those without (p < 0.0001). Study participants with larynx cancer had significantly higher mean waist circumferences, systolic and diastolic blood pressures (BP), fasting blood glucose (FBG) levels, fasting triglyceride (TG) levels, fasting total cholesterol levels, and fasting low-density lipoprotein (LDL) cholesterol levels (p < 0.0001) than those without larynx cancer.

**Table 1:** Analysis of factors potentially associated with laryngeal cancer (n = 23,253,270).

Parameter	Yes (n = 5,322)	No (n = 23,247,948)	P-value
Age (years)	63.29 ± 9.71	47.7 ± 14.4	<0.0001^*^
Gender (%)			<0.0001^*^
Male	93.16	50.69	
Female	6.84	49.31	
Smoking status (%)			<0.0001^*^
Never smoker	25.44	61.92	
Ex-smoker	26.51	13.54	
Current smoker	48.05	24.54	
Drinking level (%)			<0.0001^*^
None	41.86	53.62	
Mild	41.83	39.77	
Heavy	13.31	6.61	
Routine exercise (%)	45.1	49.58	<0.0001^*^
Diabetes (%)	20.35	9.3	<0.0001^*^
Hypertension (%)	49.4	26.36	<0.0001^*^
Body mass index (kg/m^2^)	23.38 ± 3.07	23.69 ± 3.27	<0.0001^*^
Waist circumference (cm)	84.0 ± 8.25	80.04 ± 9.27	<0.0001^*^
Systolic BP (mmHg)	128.5 ± 16.14	122.24 ± 15.19	<0.0001^*^
Diastolic BP (mmHg)	78.56 ± 10.02	76.07 ± 10.1	<0.0001^*^
Glucose (mM)	104.68 ± 27.79	97.55 ± 23.41	<0.0001^*^
Total cholesterol (mM)	194.68 ± 36.88	192.26 ± 38.20	<0.0001^*^
HDL cholesterol (mM)	53.30 ± 20.30	55.45 ± 16.69	<0.0001^*^
LDL cholesterol (mM)	113.4 ± 33.64	109.76 ± 35.68	<0.0001^*^
Triglyceride (mM)	129.80 (127.89–131.73)	116.60 (11.57–111.63)	<0.0001^*^
Metabolic syndrome (%)	38.42	69.92	<0.0001^*^

Values are mean ± SE or % ± SE.

^*^Significant at p < 0.05.

### Relationship between individual components of MetS and larynx cancer

Table 2 presents data on the relationship between larynx cancer and each individual component of MetS according to logistic regression models. The rate of age, sex, smoking status, alcohol intake, and motor coordination risk is 1.13 times higher than that of patients without MetS. Age, gender, smoking status, alcohol intake, and exercise-adjusted hazard ratios indicate that the rate of having larynx cancer with MetS is 1.13 times higher than that of patients without MetS. The adjusted multivariable model shows that elevated FBG, elevated TG, low high-density lipoprotein (HDL) cholesterol, and elevated BP were significantly associated with an increased hazard of larynx cancer.

**Table 2:** Multivariate Cox proportional hazard model for incidence of larynx cancer according to the presence or absence of each metabolic syndrome component.

Parameter	No. of patients	Person -years	Annual incidence rates	Hazard Ratio (95% confidence interval)		
				Model 1	Model 2	Model 3
Presence of metabolic syndrome						
Yes	2,190	3,52,16,370	6.2187	1.804 (1.709–1.906)	1.132 (1.071–1.196)	1.130 (1.069–1.193)
No	3,132	9,09,84,120	3.44236	1	1	1
High waist circumference						
Yes	1,449	3,57,86,984	4.04896	0.945 (0.890–1.004)	0.981 (0.923–1.043)	1.006 (0.947–1.070)
No	3,873	9,04,13,506	4.28365	1	1	1
High glucose						
Yes	2,608	4,00,57,993	6.51056	2.065 (1.957–2.179)	1.137 (1.077–1.200)	1.123 (1.064–1.186)
No	2,714	8,61,42,496	3.15059	1	1	1
High triglyceride						
Yes	2,484	4,41,28,178	5.62906	1.626 (1.541–1.716)	1.148 (1.088–1.212)	1.092 (1.035–1.153)
No	2,838	8,20,72,312	3.45793	1	1	1
Low HDL cholesterol						
Yes	1,700	3,63,33,880	4.67883	1.159 (1.094–1.128)	1.053 (0.993–1.116)	1.087 (1.025–1.153)
No	3,622	8,98,66,610	4.03042	1	1	1
High blood pressure						
Yes	3,565	5,52,50,604	6.45242	2.601 (2.457–2.754)	1.116 (1.052–1.118)	1.120 (1.056–1.189)
No	1,757	7,09,49,886	2.4764	1	1	1

Model 1: Unadjusted.

Model 2: Adjusted for age and gender.

Model 3: Adjusted for age, gender, smoking status, alcohol intake, and exercise.

### Relationship between MetS, the number of MetS components, and larynx cancer

**Figure 1:**
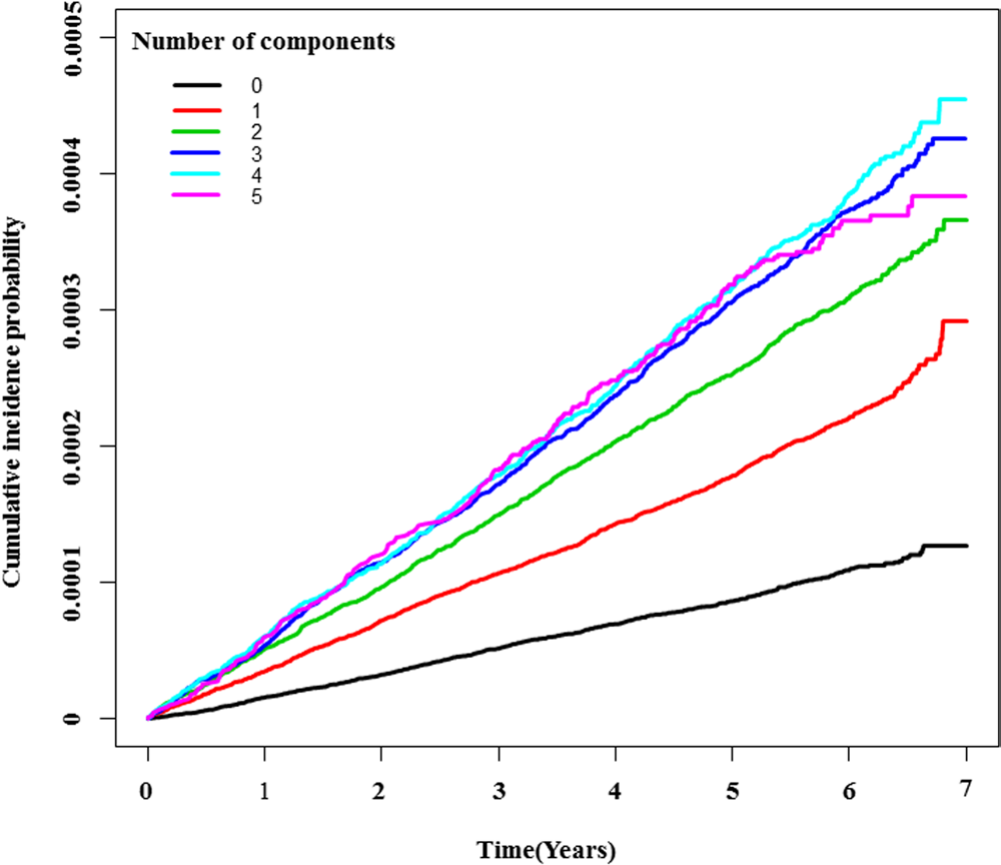
Prevalence of larynx cancer by number of metabolic syndrome components.

We examined the joint effect of smoking and the number of MetS components at baseline on the risk of laryngeal cancer (Table 3). The number of MetS components was found to be a strong risk factor, with a higher risk estimate of laryngeal cancer in people who are ex- or current smokers as well as people who have never smoked. Specifically, there was an association between smoking and the risk of laryngeal cancer among even those who had never smoked with three, four or five MetS components (hazard ratios [HR]: 1.224; 95% confidence intervals [CI]: 1.009–1.484). Figure 1 shows a significant linear trend between the prevalence of larynx cancer and the number of MetS components present.

**Table 3:** Segmented linear regression model of metabolic components for the risk of larynx cancer according to smoking status.

Smoking status	No. of metabolic components	Hazard Ratio (95% confidence interval)
Never smoker	0	1
	1,2	1.220 (1.009,1.475)
	3,4,5	1.224 (1.009,1.484)
Ex-smoker	0	1.824 (1.430,2.326)
	1,2	1.924 (1.592,2.336)
	3,4,5	2.159 (1.782,2.616)
Current smoker	0	3.170 (2.579,3.896)
	1,2	3.513 (2.924,4.222)
	3,4,5	3.983 (3.307,4.797)

Adjusted for age, gender, alcohol intake, and exercise.

### Relationship between combinations of MetS components and larynx cancer

Table 4 shows the risk of developing larynx cancer according to each of the fifteen specific combinations of MetS components after adjustment for confounders: ten combinations of three components and five combinations of four components. In multivariable analyses subjects with the following three combinations had the highest HR of having larynx cancer: (1) abdominal obesity, elevated BP, and elevated FBG (HR, 1.325; 95% CI, 1.130–1.554); (2) elevated FBG, elevated BP, and elevated TG (HR, 1.129; 95% CI, 1.055–1.409); and (3) elevated TG, elevated FBG, and low HDL cholesterol (HR, 1.357; 95% CI, 1.1073–1.664). The following four combinations had the highest HR of having larynx cancer among the five combinations of four MetS components: (1) abdominal obesity, elevated TG, elevated BP, and low HDL cholesterol (HR, 1.226; 95% CI, 1.025–1.466); and (2) elevated BP, elevated TG, elevated FBG, and low HDL cholesterol (HR, 1.337; 95% CI, 1.171–1.526).

**Table 4:** Multivariable-adjusted hazard ratio of having larynx cancer for specific combinations of metabolic components.

No. of components	Variable	Hazard Ratio (95% confidence interval)
0	None	1
3	AO + EBP + FBG	1.325 (1.130,1.554)
	AO + EBP + TRG	1.205 (0.984,1.477)
	AO + EBP + LHDL	1.026 (0.721,1.459)
	AO + FBG + TRG	1.047 (0.751,1.460)
	AO + FBG + LHDL	0.865 (0.448,1.672)
	AO + TRG + LHDL	1.274 (0.940,1.726)
	EBP + FBG + TRG	1.219 (1.055,1.409)
	EBP + FBG + LHDL	1.184 (0.917,1.529)
	EBP + TRG + LHDL	1.264 (1.09,1.467)
	FBG + TRG + LHDL	1.357 (1.107,1.664)
4	AO + EBP + FBG + TRG	1.071 (0.893,1.285)
	AO + EBP + FBG + LHDL	1.035 (0.741,1.445)
	AO + EBP + TRG + LHDL	1.226 (1.025,1.466)
	AO + FBG + TRG + LHDL	1.202 (0.893,1.619)
	EBP + FBG + TRG + LHDL	1.337 (1.171,1.526)

AO, abdominal obesity; EBP, elevated blood pressure; FBG, elevated fasting glucose; TRG, elevated triglycerides; LHDL, Low high-density lipoprotein.

Adjusted for age, gender, smoking status, alcohol intake, and exercise.

## Discussion

To our knowledge, we showed that the first time report about the associations between the presence of MetS, individual MetS components, and various combinations of MetS components and laryngeal cancer. From the result of our present study, we proved that the prevalence of laryngeal cancer among a group with MetS was significantly higher than that among a group without MetS independent of age, gender, smoking status, alcohol intake, and exercise. Smoking can cuase gender based difference for laryngeal cancer. According to the National Smoking Prevalence Survey, the adult smoking rate was 79.3 percent for males and 12.6 percent for females in Korea^14^.

Classically, cigarette smoking has been thought of the most important risk factor for laryngeal cancer. Franceschi *et al*. reported that relative to non-smokers the risk among ever-smokers for larynx cancer were 4.8 (95% CI, 2.3–10.1) for low/medium and 7.1 (95% CI, 3.2–15.6) for high tar cigarettes after adjustment for other risk factors^15^. We determined the effect of the number of the metabolic components on the risk of larynx cancer according to smoking status. Our results showed that linear associations between the number of the components of MetS and laryngeal cancer were stronger in current smoker than in ex-smoker suggesting more pronounced effects of MetS on laryngeal cancer in current smoker than in ex-smoker. A strong association with laryngeal cancer was also observed in individuals with three, four or five components, even those who were never smokers (HR: 1.224; 95% CI: 1.009–1.484). Compared with individuals presenting none of the components of MetS, relative risks were increased 3.513 (current smoker; 95% CI, 2.924–4.222), 1.924 (ex-smoker; 95% CI, 1.592–2.336) times more for individuals who had one or two metabolic components, and 3.983 (current smoker; 95% CI, 3.307–4.797), 2.159 (ex-smoker; 95% CI, 1.782–2.616) times more for those who had three, four, or five components.

A strong association has been found between the number of MetS components and the HR of laryngeal cancer. Of the three MetS components, the combination of elevated FBG, elevated TG, and low HDL was related to the highest risk of laryngeal cancer. Of the four MetS components, the combination of elevated FBG, elevated BP, low HDL, and elevated TG was related to the highest risk of laryngeal cancer. Furthermore, elevated FBG had the highest odds for larynx cancer in Table 2 after adjustment for age, gender, exercise, smoking status, and alcohol intake (HR: 1.123; 95% CI: 1.064–1.186). This pattern emphasizes the effect of diabetes on the cancer of the larynx in the Korean population. There was also a significant linear association between the number of MetS components and the probability of developing larynx cancer. Participants with five MetS components had 1.275-fold higher odds (95% CI 1.107–1.468) of having laryngeal cancer than those without MetS (HR, 1.018; 95% CI, 0.847–1.228 in one component: HR, 1.077; 95% CI, 0.842–1.393 in two components: HR, 1.177; 95% CI, 0.914–1.542 in three components: HR, 1.179; 95% CI, 0.945–1.468 in four components). Figure 1 revealed a strong association between the prevalence of laryngeal cancer and the number of MetS components present.

Based on previous literatures, Possible mechanism of carcinogenesis of MetS is presumed as belows; (1) insulin resistance and hyperinsulinemia, (2) chronic subclinical inflammation, (3) abnormalities in sex hormones metabolism, (4) damage by endocrine disruptors exposure and air pollution, (5) chronic hyperglycemia, and (6) Disorders of circadian clock^16^. In the aspect of pathophysiology of MetS, hyperglycemia is considered as a key factor. Hyperinsulinemia, insulin resistance, and hyperglycemia are increasing proliferation, angiogenesis, damage to the DNA molecule by oxygen active forms due to glucose excess, cellular mobility, and apoptosis^17–19^. From the results of previous literatures, it has been suggested that, MetS affects tumor cells cancer and finally increasing the risk of cancer through the change in insulin receptor expression as well as the activation/inactivation of the growth and transcription factors, their receptors^17,20–22^. Another notable point is the role of insulin growth factor-1 (IGF-1) and insulin receptors which are overstimulated in MetS due to insulin resistance^23^. It is known that some tumors, especially squamous cell carcinoma in lung cancer, produce high levels of IGF-1 themselves. IGF-1 usually acts as a promoting factors for stimulation of differentiation, proliferation, and protein synthesis, and at the same time it reduces the apoptosis of cells^24^. IGF-1 also regulates the cell cycle through modulation of cyclin-dependent kinases, cyclin-dependent kinase inhibitors, and cyclins^25^. It has been revealed that in osteosarcoma, gynecological, gastrointestinal, proste and lung cancers, the increasing level of IGF-1 and changes of IGF-1 signaling pathway are frequently noticed.

This study has several limitations. First, the research groups are subject to selection bias. Although all Korean residents deserve medical evaluations every two years, it is not actually mandatory but voluntary to be able to receive medical evaluations every two years. Second, the lack of detailed biochemical information including the cancer stage, physical findings and other medical history. Third, the study cohort with laryngeal cancer was small, leading to large standard deviations. And the mean age was higher in the populations with laryngeal cancer than in those without. Age differences can contribute to the association between MetS and larynx cancer. Lastly, although this study has found a statistically significant relationship between MetS and larynx cancer, we still do not have sufficient evidence to claim causality. Based on the result of this study, further investigation to figure out the pathophysiologic mechanism between MetS and laryngeal cancer is still needed.

In conclusion, this population-based study shows evidence of the association between the presence of MetS and development of laryngeal cancer, and the increases in risk vary according to the number and various combinations of MetS components. Although precise pathophysiology could not be determined from the data in our our study, the current results may be helpful for further investigations to verify the relationship between MetS and oncogenesis of laryngeal cancer.

## Materials and Methods

### Study population

The Korean National Health Insurance Service (KNHIS) is the public medical insurance system which is administered by the Ministry for Health, Welfare and Family Affairs^26^. As a compulsory social insurance system, the KNHIS program includes about 97% of the entire Korean population. The remaining 3% of the population protected by a Medical Aid program. The KNHIS database includes patient demographics and records on diagnosis, interventions, and prescriptions^27^. Diagnoses were confirmed using the International Classification of Disease, Tenth Revision, Clinical Modification (ICD-10-CM) codes. Larynx cancer was defined as C32.0–32.3, C32.8, and C32.9. Informed consent was reviewed and signed by all participants. The research protocol was approved by the Institutional Review Board of the Catholic University of Korea. Methods were performed in accordance with the relevant guidelines and regulations.

### Patients selection

People who join the National Health Insurance Corporation are encouraged to have standardized medical tests every two years. A total of 23,253,270 people who received the KNHIS health checkup in 2009 and 2010 were included in the KNHIS national sample cohort, and we followed these individuals until 2015. Participants were defined as having larynx cancer if they had admissions records for larynx cancer in their national health insurance data from 2009 to 2015. Participants were defined as larynx cancer if they had a hospitalization record for larynx cancer in the National Health Insurance data from 2009 to 2015. The medical examinations included measurements of height, weight, and BP. Levels of FBG, TG, total cholesterol, and HDL were also obtained. Health-related behaviors, such as past medical history and smoking, alcohol consumption and physical activity, were collected using standardized self-reporting questionnaires.

### Definition of metabolic syndrome

The definition of MetS was based on a definition established by the Task Force’s joint interim report for epidemiology and prevention by the IDF Special Committee^28^. According to this institution, MetS should have at least three of the following five components: abdominal obesity (≥90 cm for men and ≥85 cm for women), elevated BP (systolic ≥ 130 and/or diastolic ≥ 85 mmHg), hyperglycemia (FBG ≥ 100 mg/dL), hypertriglyceridemia (TG ≥ 150 mg/dL), and low HDL-cholesterol levels (<40 mg/dL for men and <50 mg/dL for women)^29^. The BMI was calculated by dividing the weight in kilograms by the square of height in meters (kg/m^2^).

### Statistical analysis

Statistical analyses were performed using SAS software (version 9.2; SAS Institute, Cary, NC, US). Baseline characteristics of study participants based on the presence of MetS are expressed as mean ± standard deviation for continuous variables and as a percentage of the number of categorical variables. The values were compared using independent t tests for continuous variables and chi-square tests for categorical variables. The incidence of larynx cancer was calculated by dividing the number of cases by 1,000 person-years. A Cox proportional risk analysis was performed to assess the association of the MetS and its components with larynx cancer, and hazard ratio (HR) and the 95% confidence interval (CI). Model 1 was unadjusted. Model 2 was adjusted for age and sex. Model 3 was additionally adjusted for smoking status, alcohol intake, and exercise. The Kaplan-Meier curve represents the cumulative probability of developing larynx cancer and a log ranking test was performed to investigate the association of the number of MetS components at risk of larynx cancer. We also assessed the risk of developing larynx cancer according to the number of MetS components used by individuals in the Cox proportional risk analysis. Analyzing hierarchy by gender and age was also performed. A p-value < 0.05 was considered statistically significant.
